# A Case of Rhabdomyolysis Induced by Antipsychotic Medication With Creatine Kinase (CK) Levels Elevated to 420,000 U/L, Resulting in Acute Kidney Injury (AKI) and Necessitating Hemodialysis

**DOI:** 10.7759/cureus.58145

**Published:** 2024-04-12

**Authors:** Daichi Omote, Nobuhiko Kuramoto

**Affiliations:** 1 Nephrology, Narita Red Cross Hospital, Narita-shi, JPN

**Keywords:** aki, creatine kinase, antipsychotic drugs, hemodialysis, rhabdomyolysis

## Abstract

A 45-year-old man on public welfare, who had been visiting a psychiatric hospital for schizoaffective disorder, began working as a package delivery person for the first time in the morning after receiving welfare. In the afternoon, he noticed pain in his lower back. By evening, he was unable to move, prompting an emergency call and transportation to our hospital. Blood tests revealed renal damage and elevated creatine kinase (CK) levels, resulting in hospitalization. Although he received fluid replacement after admission, he did not urinate, and his CK levels increased to 420,000 U/L, necessitating hemodialysis. Subsequently, his CK levels gradually improved over time, accompanied by increased urine output. Approximately three weeks after initiating hemodialysis, he was weaned off the treatment and discharged home 40 days after admission.

## Introduction

Rhabdomyolysis is the destruction or necrosis of skeletal muscles caused by physical or non-physical factors, resulting in the leakage of myoglobin, creatine kinase (CK), lactate dehydrogenase, aspartate aminotransferase, alanine aminotransferase, and electrolytes that compose muscle cells into the blood. When rhabdomyolysis becomes severe, the individual may be unable to urinate on their own, and hemodialysis may be required for treatment. Here, we present a case of a patient who developed rhabdomyolysis due to manual labor while taking antipsychotic drugs for schizoaffective disorder, necessitating hemodialysis. The patient underwent hemodialysis and was successfully weaned off dialysis after three weeks. Antipsychotic drugs for schizoaffective disorder were suspected as the cause of the elevated CK levels.

## Case presentation

A 45-year-old man presented with symptoms of lumbar back pain and difficulty in moving. He had a medical history of schizoaffective disorder and was using olanzapine, risperidone, quetiapine, biperiden, levomepromazine, lorazepam, nitrazepam, triazolam, and bromazepam. There was no recent change or discontinuation of antipsychotic medications; he was taking his medications well every day, and his physical condition was normal until the day before admission. He had quit his job nine months earlier and was on welfare. In an effort to make ends meet, he took a morning job as a baggage carrier as his day job for the first time since receiving welfare. In the afternoon, he noticed lower back pain, and in the evening he had difficulty moving, so an emergency call was made, and he was transported to our hospital.

Tachypnea and decreased oxygenation were noted at the time of examination; the patient was given a 10L oxygen mask. His temperature was slightly elevated at 37.1°C, but his blood pressure and pulse were normal. He complained of lumbar back pain and dysmobility, but no other physical findings were evident. There were no palpitations, abdominal pain, or leg edema, and no sweating, skin pigmentation, numbness, or muscle stiffness. Arterial blood gases showed no partial pressure of carbon dioxide (PCO2) retention, and partial pressure of oxygen (PO2) was maintained, although the patient was under 10 L of oxygen. In addition, blood tests showed mildly impaired renal function with creatinine of 1.72 mg/dL, abnormally high CK of 91904 IU/L, elevated aspartate aminotransferase of 473 IU/L, alanine aminotransferase of 93 IU/L, and lactate dehydrogenase of 3056 IU/L. Potassium was 6.8 mEq/L, and electrolytes were abnormal. White blood cells were elevated at 19800/µL (Table 1). A urine test could not be submitted because the patient did not urinate on his own, and it could not be collected even after catheterization. Imaging studies included chest radiography, which showed a cardiothoracic ratio of 57%; there was no evidence of decreased lung field permeability or pleural effusion. A simple CT examination of the chest and abdomen revealed bilateral pleural effusions but no noticeable obstruction of the urinary tract, renal enlargement, atrophy, or neoplastic lesions. An ultrasound examination and a CT scan showed that the kidney size was approximately 10 cm, which was within the normal range. Based on the clinical presentation and findings, the patient was diagnosed with rhabdomyolysis and acute kidney injury (AKI).

**Table 1:** Laboratory findings at the time of admission MB, myocardial band; eGFR, estimated glomerular filtration rate; MPO-ANCA, myeloperoxidase antineutrophil cytoplasmic antibody; PR3-ANCA, proteinase 3 antineutrophil cytoplasmic antibody; GBM, glomerular basement membrane; HBs, hepatitis B surface; HBc, hepatitis B core; HCV, hepatitis C virus; pH, potential hydrogen; PCO2, partial pressure of carbon dioxide; PO2, partial pressure of oxygen; HCO3−, bicarbonate ion

Test	Unit	
Blood cell count		
White blood cell	/μL	19800
Hemoglobin	g/dL	16.7
Platelet	/μL	290,000
Blood chemistry tests		
Aspartate aminotransferase	IU/L	473
Alanine aminotransferase	IU/L	93
Lactate dehydrogenase	IU/L	3056
Alkaline phosphatase	IU/L	168
Creatine kinase	IU/L	91904
Creatine kinase-MB	IU/L	67
Total bilirubin	mg/dL	0.4
Total protein	g/dL	8
Albumin	g/dL	4.6
Uric acid	mg/dL	25.8
Blood urea nitrogen	mg/dL	14
Creatinine	mg/dL	1.72
eGFR-creatinine	mL/min/1.73㎡	36
Sodium	mEq/L	132
Potassium	mEq/L	6.8
Chloride	mEq/L	91
Cardiac muscle troponin T	ng/mL	0.05
N-terminal-pro brain natriuretic peptide	pg/mL	133
Glucose	mg/dL	99
Hemoglobin A1c	%	5.8
Serology		
C-reactive protein	mg/dL	1.0
Rheumatoid factor	IU/mL	negative
Anti-nuclear antibody	-
MPO-ANCA	U/mL	<1.0
PR3-ANCA	U/mL	<1.0
anti-GBM antibody	U/mL	<7.0
HBs antigen	-	negative
Anti-HBs	-	negative
Anti-HBc	-	negative
Anti-HCV	-	negative
Blood gas (artery, under the administration of 10 L of oxygen)		
pH	-	7.34
PCO2	mmHg	36
PO2	mmHg	198
HCO3^-^	mEq/L	19

**Figure 1:**
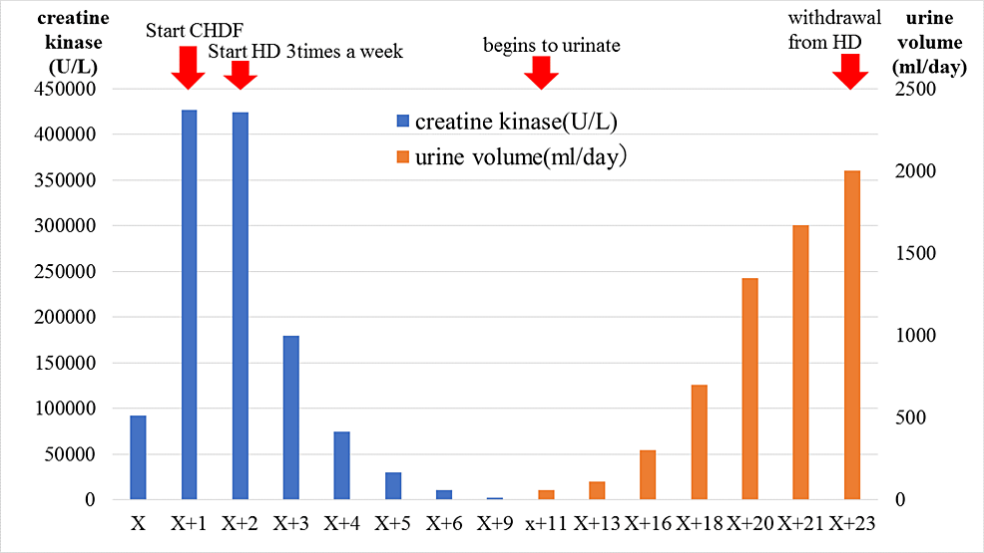
Clinical course of the present case Clinical course of the present case. Serum creatine kinase(U/L) and urine volume(ml/day) are shown. The patient was treated with hemodialysis. Serum creatine kinase gradually improved and urine volume began to increase. After three weeks of hemodialysis, he was weaned from dialysis.

After admission, he was started on high-volume replacement fluid. On Day X+1, no urine output was noted from admission to the next morning, and the blood test showed CK elevated to 426,955 U/L. He was started on continuous hemodiafiltration. On day X+2, nephrology was consulted. He was moved to hemodialysis three times a week. Due to schizoaffective disorder, he had disturbing behavior in the ward and during dialysis and was moved to the psychiatric ward on day X+6. The dosage of antipsychotic medication was adjusted in the psychiatric ward, and he was able to remain calm during his stay in the hospital, and his restless behavior during dialysis stopped. Urine production started on day X+11. Initially, the urine was reddish brown, but as time progressed, the urine changed to pale yellow. On day X+23, he was weaned from hemodialysis because his urine output was about 2000 ml/day and his creatinine level improved to 3 mg/dL on the blood test (Figure 1). Thereafter, his serum creatinine levels normalized, and he was discharged home on day X+40. His renal function did not deteriorate for more than a year after his discharge from the hospital.

## Discussion

Rhabdomyolysis is caused by the leakage of myoglobin, CK, lactate dehydrogenase, aspartate aminotransferase, alanine aminotransferase, and electrolytes from muscle cells into the blood as a result of destruction or necrosis of skeletal muscle caused by physical or non-physical factors. The large amount of leaked myoglobin may obstruct the renal tubules, resulting in AKI. There is also a risk of shock due to decreased circulating blood volume and sudden cardiac arrest due to hyperkalemia. The causes of rhabdomyolysis can be classified into physical and non-physical factors [1]. Physical factors include trauma, excessive exercise, heat stroke, and malignant syndromes [1]. Nonphysical factors include drug-related, such as hyperlipidemic drugs, antibacterial and antipsychotic medications, electrolyte abnormalities, metabolic muscle diseases, endocrine disorders, myositis, and infection [1]. Subjective symptoms of rhabdomyolysis include weakness, swelling, numbness, pain in the extremities, and reddish brown urine (myoglobinuria), which may be accompanied by anuria and oliguria as renal symptoms.

Laboratory findings in rhabdomyolysis include elevated CK, lactate dehydrogenase, aspartate aminotransferase, alanine aminotransferase, and myoglobin in blood tests. Myoglobin should be measured by urinalysis as well as blood tests. In rhabdomyolysis with AKI, blood tests often also show elevations in potassium, phosphorus, uric acid, and lactic acid. The most sensitive laboratory finding of muscle damage is an elevated serum CK level. An elevated serum CK of 5000 U/L is associated with kidney injury [1], and kidney injury is a common complication when serum CK exceeds 16,000 U/L and sometimes 100,000 U/L [2]. Around 81% of patients with serum CK > 5000 have AKI and 26% require hemodialysis [3]. Early massive infusion of fluids, hyperkalemia control and urinary alkalinization, and forced diuresis are used to treat and prevent AKI. However, for patients with complete kidney injury who are unable to urinate on their own even with the above treatments, blood purification is indicated. The mortality rate of rhabdomyolysis is approximately 10% and is even higher in cases of severe kidney injury, so early treatment is necessary ^[2]^.

 In our case, the patient was carrying luggage, which may have caused excessive muscle stress and dehydration. He was taking antipsychotic medications, and we hypothesized that in addition to physical exhaustion and dehydration, the antipsychotic medications compounded the development of rhabdomyolysis. Neuroleptic malignant syndromes during antipsychotic medication can cause high CK levels and renal dysfunction. Neuroleptic malignant syndrome is a life-threatening neurological emergency associated with antipsychotic use and is characterized by clinical symptoms such as altered mental status, muscle rigidity, hyperthermia, and autonomic nervous system symptoms. The Levenson and Caroff criteria have long been used as diagnostic criteria for neuroleptic malignant syndromes [4,5]. In the present case, the patient had elevated CK tachypnea, elevated white blood cell count, and metabolic acidosis, but no muscle stiffness, hyperthermia, disturbance of consciousness, abnormal blood pressure, tachycardia, or disturbance of consciousness. Thus, the diagnostic criteria for neuroleptic malignant syndrome were not met.

Although a diagnosis of neuroleptic malignant syndrome could not be made, antipsychotic drugs have been reported to cause rhabdomyolysis that is not related to neuroleptic malignant syndrome. Among antipsychotic drugs, quetiapine, clozapine, risperidone, and olanzapine frequently cause rhabdomyolysis [6]. Since the patient was taking quetiapine, risperidone, and olanzapine, antipsychotic medications may have contributed to a high level of CK elevation. It was also reported that approximately 10% of patients who developed rhabdomyolysis were taking antipsychotic medications, while only about 1.3% of the population was prescribed antipsychotics [6]. Patients taking antipsychotics should be careful because CK levels may become high when they develop rhabdomyolysis accompanied by renal damage. In rhabdomyolysis, if CK levels are around a few thousand U/L, dialysis is rarely necessary, but when the level increases to around tens of thousands of U/L, spontaneous urine production becomes impossible and hemodialysis is required [2].

In the present case, CK was temporarily elevated to 420,000 U/L, requiring hemodialysis for three weeks, but the patient was eventually able to withdraw. The half-life of serum CK-MM isoenzyme, CK fraction derived from skeletal muscle, is about 15 hours, and serum CK levels may reach a maximum on the second day of rhabdomyolysis onset. This may be the reason why the serum CK level in this case was also in the 90,000 range on admission and rose to 420,000 on the second day of admission. Renal damage due to rhabdomyolysis is not glomerular damage but tubulointerstitial damage, and renal damage is likely to be basically reversible. The present case shows that even with high CK levels, renal function can be restored by dialysis and by allowing the patient time to urinate on their own. Hemodiafiltration and continuous hemodiafiltration have been reported to be effective in removing myoglobin (molecular weight 17800) as blood purification therapy for rhabdomyolysis, but no randomized trials have evaluated hemodiafiltration and continuous hemodiafiltration. In this study, we confirmed that hemodialysis is also sufficient.

## Conclusions

In conclusion, this rhabdomyolysis patient had CK levels elevated to 420,000 U/L and required temporary hemodialysis due to severe renal impairment and lack of urine production, but successfully discontinued hemodialysis after three weeks of hemodialysis. Despite the significantly elevated CK level, the patient was successfully weaned off dialysis by administering hemodialysis and allowing for spontaneous urination. Patients taking antipsychotic medications who develop rhabdomyolysis may exhibit abnormally high levels of CK and should be carefully monitored.
